# Expression of vimentin alters cell mechanics, cell-cell adhesion, and gene expression profiles suggesting the induction of a hybrid EMT in human mammary epithelial cells

**DOI:** 10.3389/fcell.2022.929495

**Published:** 2022-09-19

**Authors:** Suganya Sivagurunathan, Amir Vahabikashi, Haiqian Yang, Jun Zhang, Kelly Vazquez, Dhivyaa Rajasundaram, Yuliya Politanska, Hiam Abdala-Valencia, Jacob Notbohm, Ming Guo, Stephen A. Adam, Robert D. Goldman

**Affiliations:** ^1^ Department of Cell and Developmental Biology, Feinberg School of Medicine, Northwestern University, Chicago, IL, United States; ^2^ Department of Mechanical Engineering *,* Massachusetts Institute of Technology *,* Cambridge *,* MA, United States; ^3^ Biophysics Program, University of Wisconsin-Madison, Madison, WI, United States; ^4^ Department of Engineering Physics, University of Wisconsin-Madison, Madison, WI, United States; ^5^ Department of Mechanical Engineering, University of Wisconsin-Madison, Madison, WI, United States; ^6^ Department of Pediatrics, University of Pittsburgh School of Medicine, Pittsburgh, PA, United States; ^7^ Department of Medicine, Feinberg School of Medicine, Northwestern University, Chicago, IL, United States

**Keywords:** vimentin, Twist1, desmoplakin, hybrid/partial EMT, intracellular mechanics, intercellular forces, cell-cell adhesion

## Abstract

Vimentin is a Type III intermediate filament (VIF) cytoskeletal protein that regulates the mechanical and migratory behavior of cells. Its expression is considered to be a marker for the epithelial to mesenchymal transition (EMT) that takes place in tumor metastasis. However, the molecular mechanisms regulated by the expression of vimentin in the EMT remain largely unexplored. We created MCF7 epithelial cell lines expressing vimentin from a cumate-inducible promoter to address this question. When vimentin expression was induced in these cells, extensive cytoplasmic VIF networks were assembled accompanied by changes in the organization of the endogenous keratin intermediate filament networks and disruption of desmosomes. Significant reductions in intercellular forces by the cells expressing VIFs were measured by quantitative monolayer traction force and stress microscopy. In contrast, laser trapping micro-rheology revealed that the cytoplasm of MCF7 cells expressing VIFs was stiffer than the uninduced cells. Vimentin expression activated transcription of genes involved in pathways responsible for cell migration and locomotion. Importantly, the EMT related transcription factor *TWIST1* was upregulated only in wild type vimentin expressing cells and not in cells expressing a mutant non-polymerized form of vimentin, which only formed unit length filaments (ULF). Taken together, our results suggest that vimentin expression induces a hybrid EMT correlated with the upregulation of genes involved in cell migration.

## Introduction

Intermediate filaments (IFs) are a large family of cytoskeletal proteins that are a feature of metazoans. They are encoded by 70 different genes subdivided into six classes (types I to VI) (Herrmann and Aebi, 2016) based on the homology of their conserved rod domains. The expression of IF proteins is cell type-specific and regulated based on the stages of differentiation of tissues and organs (Eriksson et al., 2009; Lowery et al., 2015). Vimentin, a type III IF protein is specific to mesenchymal and endothelial cells. In fully polymerized form, vimentin intermediate filaments (VIFs) are involved in cell motility (Mendez et al., 2010) and in protecting cells against mechanical stresses (Guo et al., 2013; Mendez et al., 2014; Patteson et al., 2019; Vahabikashi et al., 2019). Vimentin forms a homopolymer where two dimers associate laterally in anti-parallel fashion to form a tetramer and there is evidence that eight of these tetramers form a Unit Length Filament (ULF). ULFs anneal end to end and undergo a process of radial compaction to form ∼10 nm filaments (Herrmann et al., 1996; Herrmann and Aebi, 2016).

Vimentin has been associated with cell migration in numerous studies. For example, the expression of vimentin is initiated in human breast epithelial cells induced to migrate following the wounding of monolayer cultures. This expression ceases following the closure of the wound (Gilles et al., 1999). In addition, mice lacking vimentin have impaired wound healing and fibroblasts from vimentin knock out mice have reduced cell motility and lack directionality (Eckes et al., 2000, 1998). Further, disrupting the VIF network in fibroblasts with a dominant-negative vimentin_(1–138)_ reduces cell motility (Helfand et al., 2011) and a reduction in vimentin expression affects breast carcinoma cell migration (Messica et al., 2017). The transient expression of vimentin and its assembly into VIFs in MCF7 epithelial cells alters cell shape, motility and adhesion (Mendez et al., 2010).

Apart from the established roles of VIFs in cell migration and in protecting the mechanical integrity of cells, the expression of vimentin is considered to be an important marker of the epithelial-to-mesenchymal transition (EMT) (Zeisberg and Neilson, 2009). The EMT is a process whereby an epithelial cell transitions into a mesenchymal phenotype through a series of changes such as a loss of epithelial markers, a loss of apical-basal polarity, disruptions of cell-cell adhesions, alteration of cell-basement membrane attachments, rearrangements of cytoskeletal systems and increased motility and invasiveness (Kalluri and Weinberg, 2009). Vimentin expression appears to be indispensable for Slug or H-Ras-V12G-induced-EMT-associated cell migration mediated by its role in inducing the receptor tyrosine kinase Axl (Vuoriluoto et al., 2011). Indeed, VIFs act as a scaffold to recruit Slug to ERK, thereby increasing the phosphorylation of Slug (Virtakoivu et al., 2015).

Although there are numerous reports correlating the expression of vimentin with cell migration during a wide range of processes including wound repair and cancer metastasis (Ridge et al., 2022), little is known regarding the specific molecular and cellular changes brought about by vimentin expression. With the aim of investigating the changes that are caused by vimentin expression, we have established inducible MCF7 cell lines expressing either wild type (WT) vimentin, which polymerizes into mature VIFs, or a ULF mutant (Y117L) (Meier et al., 2009) form of vimentin, which can be expressed but cannot polymerize into mature VIFs. We have used these cell lines to identify potential alterations in gene expression and mechanical properties of MCF7 cells upon vimentin expression. The induction of vimentin expression and its assembly into polymerized VIFs alters cell mechanics, disrupts cell-cell adhesion, modifies cell-substrate and cell-cell forces and activates cell migratory pathways. Interestingly, the EMT specific transcription factor, *TWIST1* is upregulated only in WT vimentin expressing cells, but not in cells expressing the ULF form of vimentin.

## Materials and methods

### Cell culture

MCF7 cells obtained from the American Type Culture Collection (#ATCC HTB-22) were cultured in MEM (11095-080, Thermo Fisher Scientific, United States) supplemented with fetal bovine serum (Cytiva Hyclone, Fisher Scientific, United States), 50U/ml of Penicillin and 0.05 mg/ml of Streptomycin (P4333, Sigma-Aldrich, St. Louis, United States), MEM non-essential amino acids (25-025-CI, Corning, United States), sodium pyruvate (25-000-CI) and 0.01 mg/ml bovine insulin (I6634, Sigma-Aldrich, St. Louis, United States). The cells were maintained at 37 °C with 5% CO_2_.

### Constructs

WT and ULF mutant vimentin were cloned in the cumate inducible vector–pCDH-CuO-MCS-IRES-GFP-EF1α-CymR-T2A-Neo (QM812B-1, System Biosciences, United States) and transfected into 293FT packaging cells with psPAX2 (Addgene plasmid #12260) and pVSV-G (Clontech, United States) using Xfect transfection reagent (Takara Bio, United States). psPAX2 was a gift from Didier Trono (Addgene plasmid # 12260; http://n2t.net/addgene:12260; RRID:Addgene_12260) MCF7 cells were transduced with the viral particles packaged by the 293FT cells following collection of the culture medium 48 h and 72 h post transfection. Polybrene (8 μg/ml) was added to the viral particles before transducing the MCF7 cells. The transduced cells were selected with 1,400 μg/ml G418 for 14 days. Cells expressing WT vimentin that formed networks of VIFs as assessed by epifluorescence imaging, were selected by limited dilution to establish structurally homogenous populations. Cells expressing either WT or ULF mutant vimentin were induced for 5 days with 5X cumate, sorted for GFP expression and made into a homogeneous population (Supplementary Figure S1A). The sorted and selected MCF7 cells were grown with or without 5X cumate (inducer) (QM150A-1, System Biosciences, United States) and used in all experiments.

### Immunofluorescence

Cells were seeded on #1.5 coverslips and allowed to grow for at least 24 h. Subsequently the cells were fixed with 4% paraformaldehyde in PBS for 10 min at room temperature (RT) and were permeabilized for 10 min with 0.1% Triton-X 100 (Sigma-Aldrich, St. Louis, United States). After washing with 1X PBS, the coverslips were incubated for 30 min with specific antibodies diluted in 1X PBS containing 5% goat serum. The antibodies used were chicken anti-vimentin (1:200, CPCA-Vim, EnCor Biotechnology, United States), mouse anti-cytokeratin18 (1:200, PRO-61028, RDI), rabbit anti-desmoplakin (1:20, in house antibody (Jones and Goldman, 1985)), tubulin (1:500, in house antibody (DM1α)), vinculin (1:200, V4505, Sigma-Aldrich, St. Louis, United States), E-cadherin1 (1:200, M106, Takara, United States, kind gift from Cara Gottardi, Northwestern University). The coverslips were washed twice with 0.05% Tween 20 for 5 min each and further incubated with their respective secondary antibodies conjugated with Alexa Fluor dyes in 1X PBS (1:400, A-21202, A-21206, A-11041, ThermoFisher Scientific, United States). For staining actin, phalloidin (1:400, A12380, ThermoFisher Scientific, United States) was added along with the secondary antibody. The coverslips were washed twice with 0.05% Tween 20 in 1X PBS before mounting with ProLong Glass antifade mountant (P36980, Thermo Fisher Scientific, United States).

### Microscopy

Confocal images were captured with a laser scanning confocal microscope (Nikon A1R confocal microscope, Nikon, Tokyo, Japan) using an oil immersion objective lens (Plan Apo 60X Oil objective, 1.4NA, Nikon). Maximum intensity projections of the Z stacks are presented. 3D Structured Illumination Microscopy (SIM) was carried out with a Nikon N-SIM Structured Illumination Super-resolution microscope system (Nikon N-SIM, Nikon, Tokyo, Japan) using an oil immersion objective lens (CFI SR Apochromat 100X, 1.49 NA, Nikon). Raw SIM images were reconstructed with the N-SIM module of Nikon Elements Advanced Research using the following parameters: Illumination contrast:1.00; high-resolution noise suppression:0.75; out-of-focus blur suppression:0.25. Brightness and contrast were adjusted for image presentation.

Images of keratin intermediate filament (KIF) networks were analyzed for the number of junctions and keratin bundle thickness using the BoneJ plugin in ImageJ as used previously by Laly et al (Laly et al., 2021). Regions of interest with an area of approximately 85 μm^2^ were analyzed for keratin bundle thickness using the thickness option in the BoneJ plugin. The images were then binarized using the Shanbag threshold method and skeletonized. Skeletonized images were analyzed for the number of junctions detected in the KIF networks prior to and following the induction of vimentin expression.

Cells stained with phalloidin were thresholded using the ‘Huang white’ method for analyzing the aspect ratio, circularity, cell height and cell area.

Confocal images of desmoplakin across cell groups were used for the quantification of cells with disrupted desmosomes. The cells in each field were counted and considered as the total number of cells and the number of cells lacking the peripheral localization of desmoplakin were counted as cells with disrupted desmosomes. Cells with mislocalized desmoplakin even on one side of the periphery were still counted as cells with disrupted desmosomes. Percentages were calculated for the images across different cell groups.

### Western blot analyses

Cells grown in culture dishes were trypsinized and centrifuged at 1,500 rpm for 3 min. Cell pellets were lysed with Radio-Immunoprecipitation Assay buffer and sonicated. The lysates were centrifuged at 9300xg for 5 min to remove debris. The protein concentration in the supernatant was determined with the Pierce BCA protein assay kit (23225, ThermoFisher Scientific, United States). 30–50 µg protein was boiled for 10 min with the addition of 4X Laemmli buffer. The boiled lysates were electrophoresed in 10% SDS-PAGE gels and transferred to nitrocellulose membranes. The blots were blocked with 5% non-fat dry milk in 1X TBST before incubating overnight with respective antibodies–vimentin (1:1,000, CPCA-Vim, EnCor Biotechnology), Keratin8 (1:1,000, 1,432-1, Epitomics, United States) and GAPDH (1:1,000, sc-365062, Santa Cruz Biotechnology, United States). After washing the blots three times for 5 min each with 1X TBST, they were incubated for 1 h with fluorescent secondary antibodies in 1X TBST (1:10,000, IRDye 800CW, 680RD, LI-COR Biosciences, United States). The blots were then imaged with an Odyssey Fc Imaging System (LI-COR Biosciences, United States) after washing for 3X (5 min) with 1X TBST.

### RNA sequencing library preparation

RNA from cell lines was isolated using the RNAeasy Plus mini kit (Qiagen, Germany). RNA quality and quantity were measured using Agilent 4,200 Tapestation with the high Sensitivity RNA ScreenTape System (Agilent Technologies). Briefly, mRNA was isolated from 80 ng of purified total RNA using oligo-dT beads (New England Biolabs, Inc). An NEBNext Ultra™ RNA kit (New England Biolabs, United States) was used for full-length cDNA synthesis and library preparation. Libraries were pooled, denatured and diluted, resulting in a 2.0 p.m. DNA solution. PhiX control was spiked at 1%. Libraries were sequenced on an Illumina NextSeq 500 instrument (Illumina Inc) using NextSeq 500 High Output reagent kit (Illumina Inc) (1x75 cycles) with a target read depth of approximate (5–10) million aligned reads per sample.

### RNA analysis

Quality controlled FASTQ files were aligned to the Ensembl *Homo sapiens* genome (GRCh38) using STAR aligner (version 2.6.1). We then used featureCounts (Liao et al., 2014) to generate counts of reads uniquely mapped to annotated genes using the GRCh38 annotation gtf file (Anders et al., 2015). Differential gene expression analysis between the different conditions was estimated using DESeq2 (Love et al., 2014) based on the negative binomial distribution. The resulting *p*-values were adjusted using the Benjamini and Hochberg’s approach for controlling the false discovery rate, and differentially expressed genes were determined at the 5% threshold. Gene set enrichment analysis was used to assess the statistical enrichment of gene ontologies, and pathways (Subramanian et al., 2005), and visualized using Clusterprofiler (v3.16.1) (Yu et al., 2012). All statistical analyses were performed using R software 4.0.1 (R Core Team (2020)).

### Real time PCR

RNA from the various cell lines was isolated using the RNAeasy Plus mini kit (Qiagen, Germany) and converted to cDNA using an iScript cDNA synthesis kit (1708891, Bio-Rad Laboratories, United States). Primers against *SNAI1* (Forward primer (FP) - GCG​AGC​TGC​AGG​ACT​CTA​AT; Reverse primer (RP)–GGACAGAGTCCCAGATGAGC), *TCF7* (FP–ACATGCAGCTATACCCAGGC; RP- CTT​GGT​GCT​TTT​CCC​TCG​AC), *TWIST1* (FP-CTCGGACAAGCTGAGCAAGA; RP- GCT​CTG​GAG​GAC​CTG​GTA​GA), *CDH11* (FP–CCCAGTACACGTTGATGGCT; RP - AAT​GAA​TTC​CGA​CGG​TGG​CT) and MMP16 (FP–TCAGCACTGGAAGACGGTTG; RP - AAA​TAC​TGC​TCC​GTT​CCG​CA) (Integrated DNA Technologies, United States) were used to amplify the respective genes with Light cycler 480 SYBR Green I master (04 887 352 001, Applied Biosystems, United States) in a CFX96 Real Time System (Bio-Rad Laboratories, United States). 18S rRNA was used as the internal control and the fold change values were calculated according to the 2^-^ ΔΔCq method (Livak and Schmittgen, 2001).

### Polyacrylamide substrates and micropatterning

Polyacrylamide gels were used as substrates for traction force microscopy as reported previously (Saraswathibhatla and Notbohm, 2020). The gels were made with a Young’s modulus of 6 kPa and thickness of 100 µm embedded with 0.008% (weight/volume) fluorescent particles (diameter 0.2 µm, carboxylate modified; Life Technologies). The fluorescent particles were localized to the top surface of the gels by centrifuging upside down during polymerization at 700 rpm for 10 min. Polydimethylsiloxane sheets with 1.0 mm diameter holes were placed on top of the gels, enabling functionalization of 1-mm circular islands of collagen I (1 ml of 0.1 mg/ml) using sulfo-SANPAH. For each dish, 500 µL of cell solution (0.6 million cells per mL of medium) was pipetted onto the masks and incubated at 37°C for 2 h. After the sheets were removed, confluent cell islands formed within 10–12 h and were subsequently imaged.

### Imaging for traction force microscopy and monolayer stress microscopy

An Eclipse Ti microscope (Nikon, Melville, NY) with a 10× numerical aperture 0.5 objective (Nikon) and an Orca Flash 4.0 camera (Hamamatsu, Bridgewater, NJ) running Elements Ar software (Nikon) was used for imaging. Images were captured of both the cells (by phase contrast) (1 frame) and the fluorescent particles (50 frames). During imaging, the cells were maintained at 37°C and 5% CO_2_. After imaging, cells were removed by treating with 0.05% trypsin for 1 h at 37°C, and reference images of the fluorescent particles were collected.

### Fluctuation based super resolution processing

FBSR processing was performed using NanoJ-LiveSRRF (Gustafsson et al., 2016; Stubb et al., 2020). Each set of images of fluorescent particles (50 frames at a resolution of 2048 × 2044 pixels with 0.65 µm/pixel) was used for reconstruction to increase the spatial resolution by a factor of two (4,096 × 4,088 pixels with 0.325 µm/pixel). The optimal reconstruction parameters were defined by resolution-scaled error (RSE) and resolution-scaled Pearson coefficient (RSP), computed by LiveSRRF.

### Traction force microscopy and monolayer stress microscopy

Traction refers to the vector field of in-plane force per area applied by the cells to the substrate. To quantify tractions, cell-induced displacements of the fluorescent particles were measured using Fast Iterative Digital Image Correlation (FIDIC) (Bar-Kochba et al., 2015) using 128 × 128 pixel (41.6 × 41.6 µm^2^) subsets centered on a grid with a spacing of 32 pixels (10.4 μm). The large subset size used reduced the noise floor substantially, which was essential for the experiments, as the cells produced small tractions. Tractions and intercellular stresses were computed using unconstrained Fourier-transform traction microscopy (Butler et al., 2002) accounting for the finite substrate thickness (del Álamo et al., 2007; Trepat et al., 2009) and monolayer stress microscopy (Tambe et al., 2013, 2011), which is based on the two-dimensional balance of forces. The root mean square of the traction vector was computed for each cell island. The two normal stresses computed from monolayer stress microscopy were averaged to quantify the tension; the mean tension was computed for each cell island.

### Measurement of intracellular motion

All samples were imaged on a Leica SP8 confocal microscope with a resonant scanner at 37°C. Carboxylate-modified latex beads with a diameter of 0.5 um (Sigma-Aldrich, L3280) were added to the culture media (1:3000) 24 h prior to the measurement. Motions of endocytosed particles inside cells were recorded every 12 ms for 20 s. Particles in 10 different cells in each group were measured. All tested ULF mutant vimentin expressing cells and WT vimentin expressing cells had visible GFP. The whole experiment was repeated with a new batch of cells. Particle tracking was performed with TrackMate (Tinevez et al., 2017).

### Calculation of cytoplasmic shear modulus from intracellular motion

The complex modulus of cytoplasm follows a weak power law (Gupta and Guo, 2017).

$$G\left(\omega \right)=G_{0}{\left(-i\omega \right)}^{\beta }$$

β∼0

For a tracer particle embedded in three-dimensional matrix, the complex shear modulus of matrix and *N*-dimensional mean square displacement (MSD) of trace particles inside are related by (Mason and Weitz, 1995; Squires and Mason, 2009).

$$\langle \Delta r^{2}\left(\omega \right)\rangle =\frac{2Nk_{B}T}{3i\omega \pi dG\left(\omega \right)}$$

Combining these two relationships we get

$$\langle \Delta r^{2}{\left(\omega \right)\rangle }_{2D}=\frac{4k_{B}T}{3\pi dG_{0}}{\left(-i\omega \right)}^{-1}$$

In the time domain

$$\langle \Delta r^{2}{\left(\tau \right)\rangle }_{2D}=\frac{4k_{B}T}{3\pi dG_{0}}$$

Thus, the compliance $\left(1/G_{0}\right)$ can be estimated from the high frequency MSD of intracellular motion by

$$\frac{1}{G_{0}}=\frac{3\pi d\langle \Delta \rangle r^{2}{\left(\tau \right)}_{2D}}{4k_{B}T}$$

This relation quantifies the high-frequency equilibrium mechanics of cytoplasm and can be applied to the plateau of intracellular MSD (Gupta and Guo, 2017). In the calculation, we used MSD of the minimal time interval $\Delta r^{2}\left(\tau =12\;ms\right)$ to reflect the level of plateau. Outliers are removed. Outliers are defined as elements more than three scaled mean absolute deviation from the median.

**Statistical Analyses.** One-way ANOVA was used to compare the differences between uninduced cells, induced cells expressing WT vimentin and induced cells expressing ULF mutant vimentin. The Dunnett test was used for corrections of multiple comparisons.

### Data availability statement

The sequencing data has been deposited in Gene Expression Omnibus (Accession number – GSE206724).

## Results

### The different stages of vimentin assembly in MCF7 cells

**FIGURE 1:**
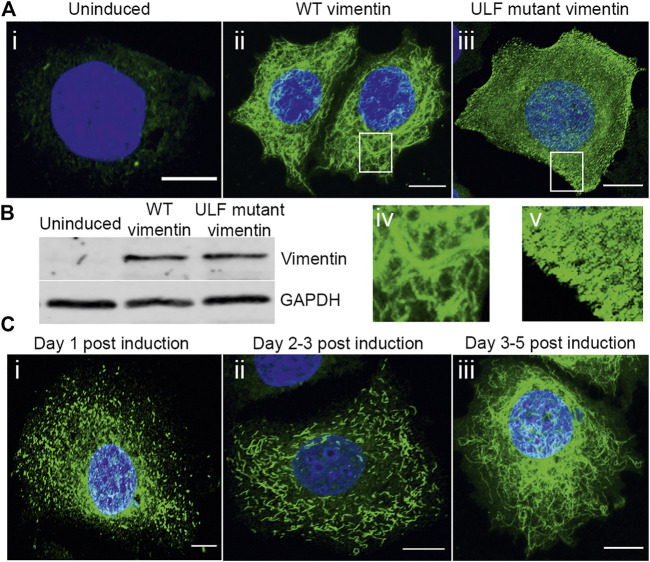
Expression and assembly stages of vimentin in inducible MCF7. **(A)** Immunofluorescence images of vimentin expression in **(A)** (i) uninduced MCF7 and (ii) cumate-induced MCF7 cells expressing WT vimentin and (iii) ULF mutant vimentin. Magnified images of WT vimentin or ULF mutant expressing cells are shown in (iv) and (v) respectively. **(B)** Western blot showing the expression levels of vimentin in uninduced MCF7 cells, WT vimentin in MCF7 cells following induction and ULF mutant vimentin in MCF7 cells, run along with an internal control, GAPDH. **(C)** Immunofluorescence images of vimentin in cumate-induced MCF7 cells expressing WT vimentin. C (i) and C (ii) show the vimentin on day 1 (C-i) and day 2 (C-ii) post cumate induction as particles and squiggles respectively, indicating the assembly stages of vimentin. c-iii is observed on day 5 post cumate induction where vimentin is observed as an extensive filamentous network. Scale bar is 10 μm.

The microinjection or transient transfection of vimentin into epithelial cells null for this type III IF protein results in rapid changes in cell shape, motility and cell-cell adhesion (Mendez et al., 2010). With the aim of understanding some of the mechanistic changes brought about by vimentin expression in tumor-derived human breast epithelial cells null for vimentin expression, MCF7 cell lines with inducible vimentin expression were established. MCF7 was chosen as the cell of choice since it expresses only an endogenous keratin IF (KIF) network and is null for vimentin. MCF7 cells were transduced with a cumate inducible vector expressing either wild type (WT) vimentin or the Y117L mutant of vimentin (Herrmann and Aebi, 2016). The Y117L mutant vimentin halts assembly at the Unit Length Filament (ULF) stage and thus provides the opportunity to express vimentin but not mature VIF networks. Addition of inducer to the cells expressing WT vimentin resulted in the assembly of a complex VIF network after 4–5 days (Figure 1 A-ii, A-iv), whereas cells expressing the ULF mutant produced only non-filamentous particles and occasional short VIFs (squiggles) (Prahlad et al., 1998) after this time period (Figures 1 A-iii, A-v). We also followed the assembly of VIFs at earlier times after the induction of vimentin expression. At 1–2 days post induction, vimentin was present primarily as particles and squiggles of short IFs (Figures 1 C-i & C-ii) and by day 5, most cells had an extensive VIF network (Figure 1C-iii), demonstrating that the WT vimentin assembled according to previously defined steps in VIF polymerization *in situ* (Prahlad et al., 1998). Immunoblotting revealed that WT and ULF mutant vimentin cells expressed similar levels of vimentin after induction (Figure 1B), while uninduced cells lacked vimentin expression (Figures 1A-i,B). After the introduction of vimentin in the MCF7 cells, we examined the cell groups for changes in the cell height, area and shape, all of which were not altered significantly between the cell groups (Supplementary Figure S1B–E).

### Expression of vimentin and the assembly of VIFs does not alter keratin expression levels but does alter the organization of keratin intermediate filaments (KIFs) in MCF7 cells

**FIGURE 2:**
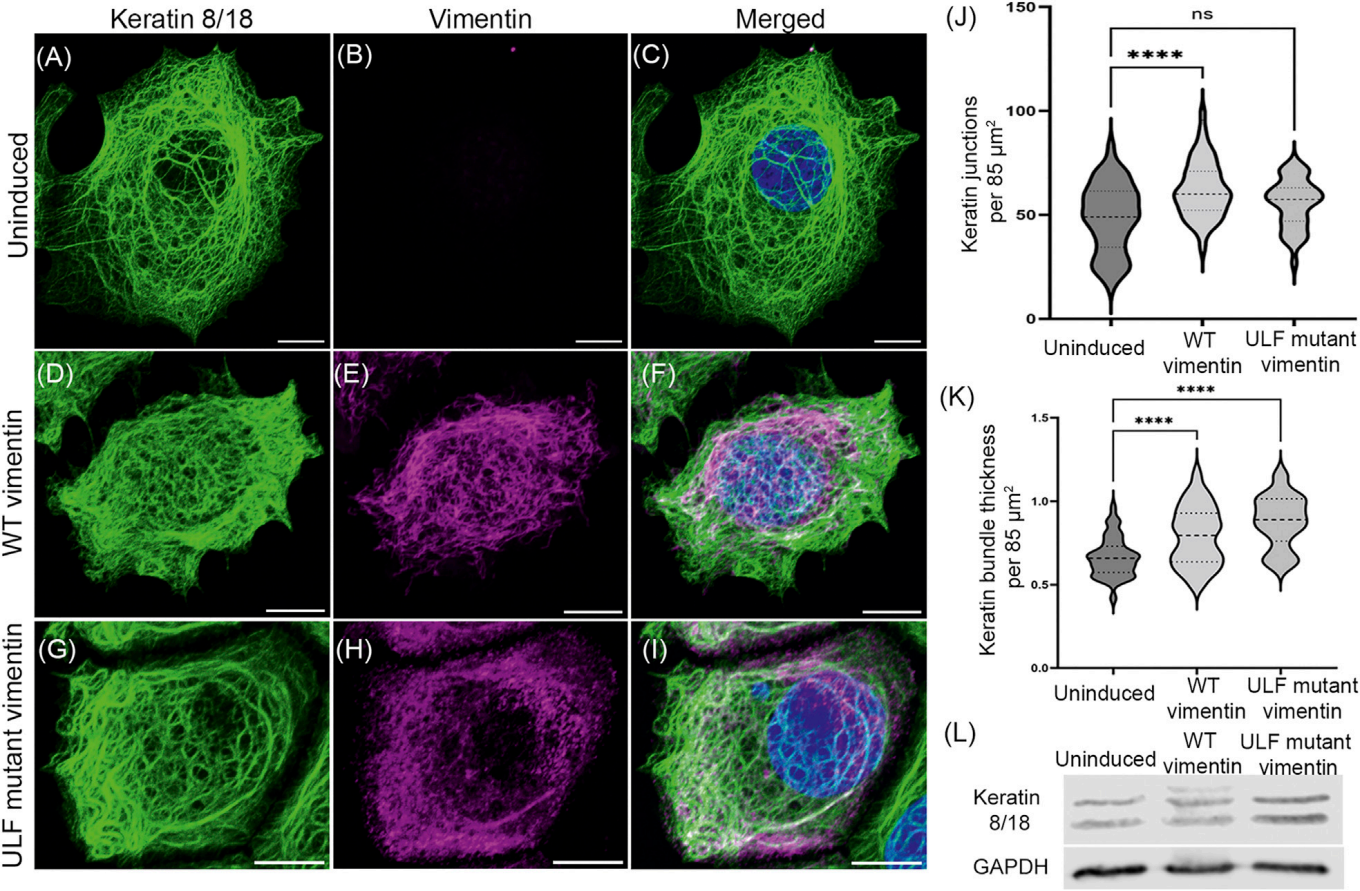
Vimentin expression does not alter the endogenous expression of keratin in MCF7. Immunofluorescence images of keratin 18 (A, D and G) and vimentin (B, E and H) in uninduced MCF7 cells **(A–C)**, WT vimentin in induced MCF7 cells **(D–F)** and ULF mutant vimentin in MCF7 cells **(G–I)**. Figures C, F and I show the overlay of keratin 18 and vimentin in the respective cells. Scale bar is 10 μm. **(J)** Graph showing the number of keratin junctions in uninduced MCF7 and in cells expressing either WT vimentin or ULF mutant vimentin. **(K)** Graph showing the keratin bundle thickness in uninduced MCF7 cells and following the induction of WT vimentin or ULF mutant vimentin. **(L)** Western blot showing the expression levels of keratin 8/18 in uninduced MCF7, MCF7 cells expressing WT vimentin or ULF mutant vimentin. GAPDH is used as loading control. One-way ANOVA was used to compare the differences between the groups. ****p < 0.0001. All the experiments were done on cells sorted for GFP expression 5 days post induction with cumate.

Cells undergoing an EMT lose their epithelial markers, including the types I and II keratins, K8/18, as they start acquiring mesenchymal features such as the initiation of vimentin expression (Zeisberg and Neilson, 2009). Therefore, we determined whether the expression levels of K8/K18 were altered after inducing vimentin expression in MCF7 cells. The results of Western blot analyses showed that the amounts of K8/K18 did not change when either WT or ULF mutant vimentin were expressed. However, we could observe an additional keratin band only in the lysates from cells expressing WT vimentin. This could possibly be attributable to a post-translational modification (Figure 2L).

The organization of K8/18 KIF networks was affected in the cells expressing WT or ULF mutant vimentin (Figures 2A–J). Specifically, morphometric analysis revealed that WT vimentin expressing cells showed a significant increase in the number of overlaps or junctions of the bundles (tonofibrils) comprising the KIF networks resulting in tighter meshworks. The tonofibrils in these cells were significantly thicker (Figures 2J,K). In contrast the number of KIF junctions was not altered in ULF mutant vimentin expressing cells but the tonofibrils were thicker when compared with uninduced cells (Figures 2J,K).

Since reorganization of cytoskeletal proteins is considered as one of the molecular processes associated with initiation of EMT (Kalluri and Weinberg, 2009), we examined the organization of actin and microtubules upon vimentin expression. Interestingly, the cortical actin was more prominent in the uninduced cells which is not the case with the cells expressing vimentin. Moreover, the cells with either form of vimentin (WT or ULF mutant) were observed to have small protrusions unlike the uninduced cells (Supplementary Figure S2). We did not observe major changes in the organization of microtubules among the cell groups (Supplementary Figure S3).

### Vimentin expression disrupts the organization of desmosomes involved in cell-cell adhesion in MCF7 cells

**FIGURE 3:**
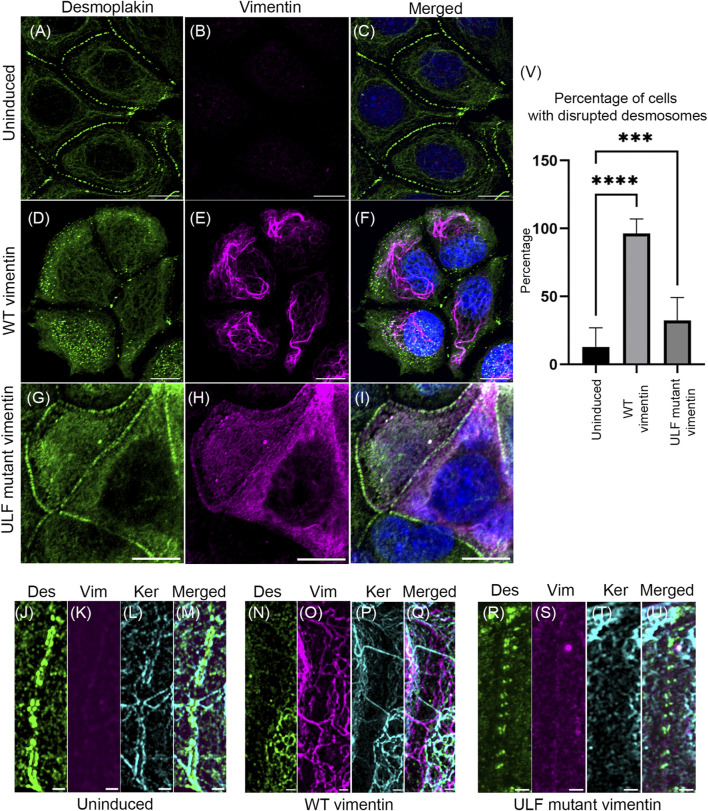
Expression of vimentin in MCF7 disrupts desmosomes. Immunofluorescence images of desmoplakin **(A,D,G)** and vimentin **(B,E,H)** in uninduced MCF7 **(A,B,C)**, cumate-induced MCF7 expressing WT vimentin **(D,E,F)** or ULF mutant vimentin **(G,H,I)**. Figures C, F and I show the overlay of desmoplakin and vimentin in the respective cells. Scale bar is 10 μm. Structured Illumination Microscopy (SIM) images of desmoplakin (J, N, R), vimentin (K, O, S), and keratin (L, P, T) in uninduced MCF7 cells **(J–M)**, cumate-induced MCF7 cells expressing WT vimentin **(N–Q)** and cumate-induced MCF7 cells expressing ULF mutant vimentin **(R–U)**. Figure M, Q and U show the respective overlays. Scale bar is 1 μm. **(V)** Graph showing the percentage of cells having disrupted desmoplakin in uninduced MCF7 cells (N = 205) and in MCF7 cells induced to express either WT (N = 101) or ULF mutant vimentin (N = 191). One way ANOVA was used to compare the differences between the groups.****p < 0.0001; ***p < 0.001. All the experiments were done on cells sorted for GFP expression 5 days post induction with cumate (Des–desmoplakin; Vim–vimentin; Ker–keratin).

Loss of cell-cell adhesion is one of the hallmarks of epithelial cells undergoing the EMT. Desmoplakin is a major component of the cytoplasmic face of the desmosomes involved in connecting KIFs to sites of cell-cell adhesion (Green et al., 1987; Smith and Fuchs, 1998). We have previously shown that introducing vimentin into MCF7 cells by transient transfection leads to loss of desmosomal structure and internalization of desmoplakin (Mendez et al., 2010). In the vimentin inducible MCF7 cells, desmoplakin was aligned in the periphery of uninduced contacting cells (Figures 3A–C). Following induction of vimentin expression, the desmosomes were disrupted and desmoplakin was displaced from the cell surfaces (Figures 3D–F). In contrast, the number of cells with disrupted desmosomes was reduced in ULF mutant vimentin expressing cells when compared to cells expressing WT vimentin (Figures 3G–I). The changes in desmosomes were revealed at higher resolution using Structured Illumination Microscopy (SIM). This demonstrated the parallel alignment of desmoplakin on either side of the surface of contacting uninduced cells (Figure 3J–M). SIM images of WT vimentin expressing cells clearly demonstrated that the desmoplakin was no longer aligned in the periphery of the majority of cells (Figure 3N–Q). Most of the mislocalized desmoplakin was associated with KIFs with a few associated with VIFs in the induced cells expressing WT vimentin (Supplementary Figure S4). In contrast, cells expressing ULF mutant vimentin largely retained desmoplakin localization in regions of cell-cell contact. However, we observed fewer linkages of keratin to desmoplakin compared to the uninduced cells (Figure 3R–U). Morphometric analysis demonstrated that desmosomes were disrupted in ∼96% of the cells expressing WT vimentin whereas only ∼32% were disrupted in cells expressing ULF mutant vimentin (Figure 3V). Overall, the results show that the expression of vimentin disrupts desmoplakin positioning at cell-cell adhesive junctions thereby causing the loss of desmosomal structures in MCF7 cells.

An important marker of the loss of cell-cell adhesion during the EMT is E-cadherin (Kalluri and Weinberg, 2009). Therefore, we determined the localization of E-cadherin in the cells with and without vimentin. Our results showed that the localization of E-cadherin appeared the same in uninduced and induced cells expressing either WT vimentin or ULF mutant vimentin (Supplementary Figure S5).

### Vimentin expression reduces both cell-substrate traction force and intercellular tension in MCF7 cells

**FIGURE 4:**
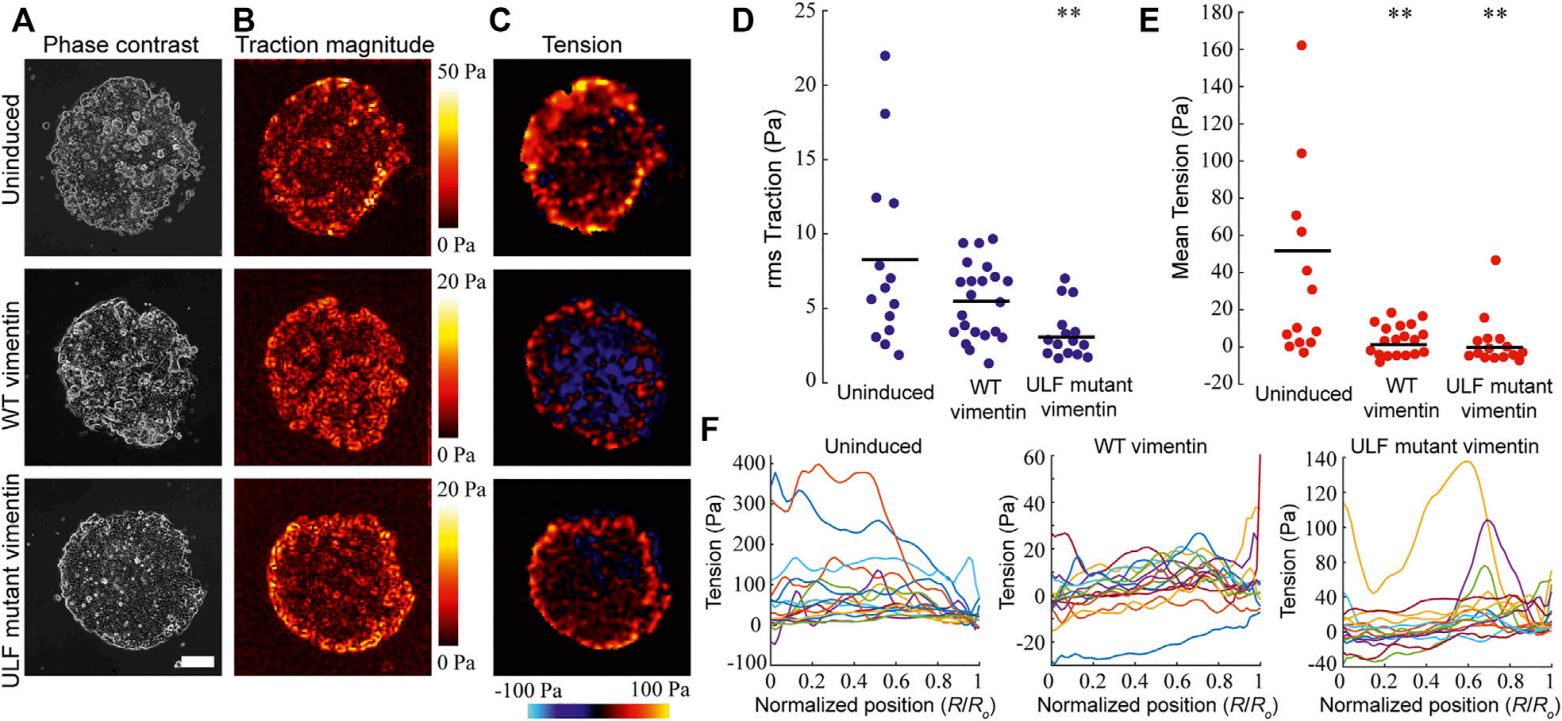
Vimentin expression reduced traction and intercellular forces in MCF7. **(A)** Representative phase contrast images of individual islands of uninduced MCF7 cells, and cumate-induced MCF7 cells expressing either WT vimentin or ULF mutant vimentin (scale bar 200 μm). **(B)** Color maps of magnitude of cell-substrate traction of cell islands corresponding to the images in column **(A)**. **(C)** Color maps of intercellular tension of the cell islands **(D)** Root-mean-square (rms) traction among uninduced MCF7 cells (*n* = 14) and MCF7 cells expressing either WT vimentin (*n* = 21) or ULF mutant vimentin (*n* = 15). **(E)** Average of intercellular stress comparison among uninduced MCF7 cells (*n* = 14), cumate-induced MCF7 cells expressing WT vimentin (*n* = 21) or ULF mutant vimentin (*n* = 15). In panels D and E, each dot represents an independent cell island, horizontal lines indicate means, and ** indicates *p* < 0.01 compared to uninduced (ANOVA with Bonferroni correction). **(F)** Intercellular stress plotted against normalized radial position, where 0 indicates the center of the cell island and *R*_0_ is the radius of the cell island. Each line represents a different cell island.

The loss of desmosomal structures following vimentin induction should lead to a weakening of the adhesive forces between adjacent cells in an epithelial sheet (Broussard et al., 2017). Since desmosomes are disrupted in the vimentin expressing cells (Figure 3), we measured the intercellular tension between uninduced and induced cells expressing WT or ULF mutant vimentin. Cells were seeded in islands of 1 mm diameter onto 3 kPa polyacrylamide gels containing fluorescent beads. The tractions and stresses were calculated using monolayer traction force and monolayer stress microscopy respectively (Figures 4A–C). The overall traction was significantly reduced in the cells expressing ULF mutant vimentin, however the reduction of traction observed in WT vimentin expressing cells did not have statistical significance compared to the uninduced cells (Figure 4D). In addition, monolayer stress microscopy indicated that the mean tension between cells expressing either WT or ULF mutant vimentin was significantly reduced compared to the uninduced cells with no significant difference between the two groups (Figure 4 C and E). It is interesting to note that although uninduced cells and the WT vimentin expressing cells exerted traction on the substrate that is not statistically different, the force was not transmitted to the neighboring cells upon vimentin expression (Figure 4 D and E). Further, we also analyzed the distribution of intercellular forces within the cell island. This was done by averaging the tension for all data points at different distances R from the center of the island. The results indicated that the intercellular tension sometimes appeared to increase with increasing radial position, but the increase was relatively small, ∼10 Pa (Figure 4F), which was smaller than the differences between uninduced cells and cells expressing either WT or ULF mutant vimentin (∼50 Pa, Figure 4E). Overall, expression of either form of vimentin reduces the cell-substrate and cell-cell forces in epithelial cells.

As indicated above, the reduction in cell-cell adhesive forces can be attributed to a loss of the structural integrity of desmosomes (Figure 3). To understand if the decreases in cell-substrate forces might be due to the alterations in cell-matrix junctions/focal adhesions, we stained the cells with vinculin and β1 integrin antibodies. Surprisingly, we did not observe significant changes in the organization of either focal adhesions or β1 integrin (Supplementary Figure S6,S7).

### Vimentin expression increases intracellular stiffness in MCF7 cells

**FIGURE 5:**
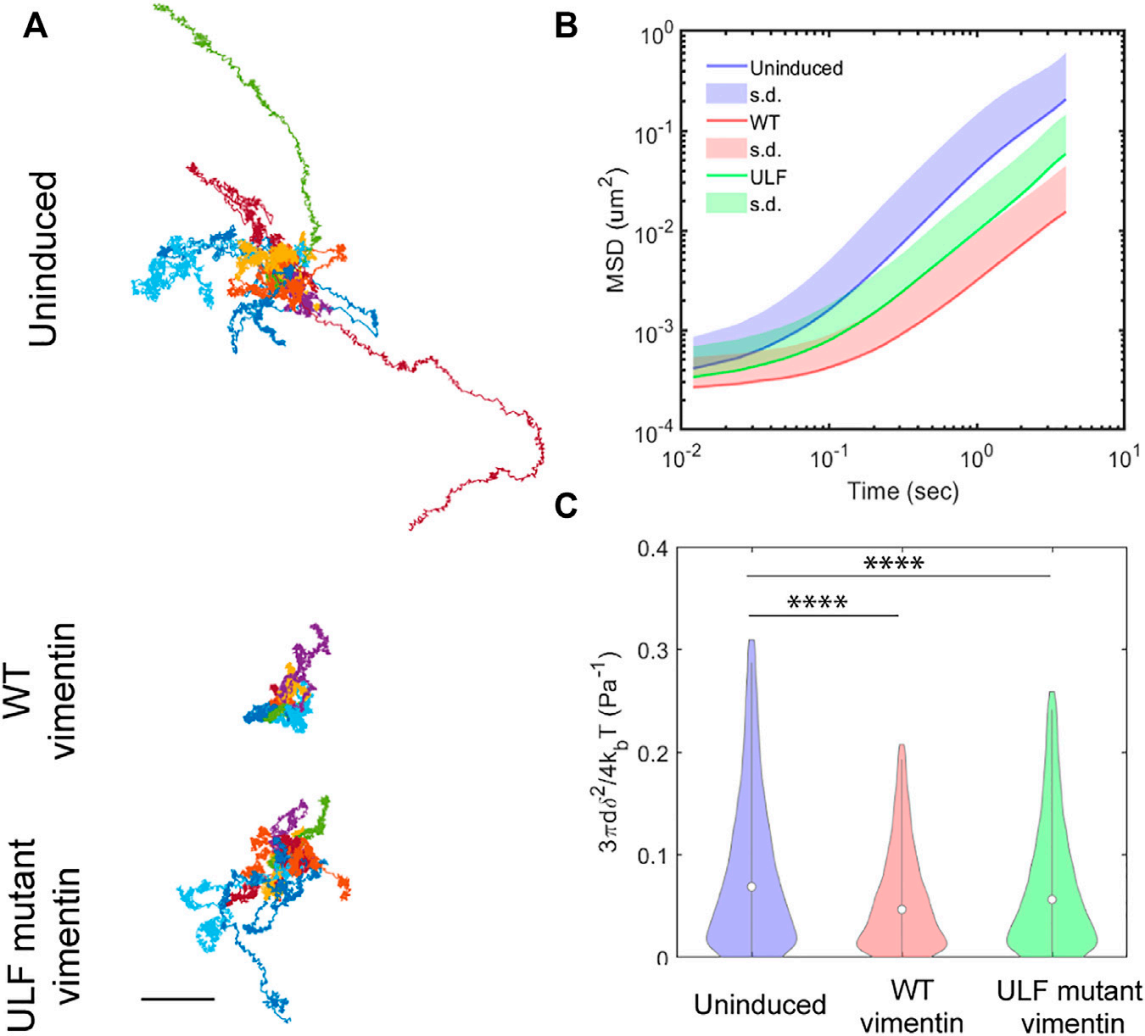
Vimentin expression reduces intracellular motion. **(A)** Trajectories of particles measured in 10 different cells in each group, from videos taken every 12 ms over 20 s time intervals. Scale bar, 0.5 µm. **(B)** Mean Square Displacement (MSD) of particles. **(C)** Compliance of cytoplasm. $\delta^{2}$ is the MSD over 12 ms, $k_{b}$ is Boltzmann constant, T= 310 K is temperature, d=0.5 µm is the diameter of the particles. One way ANOVA was used to compare the differences between the groups. ****p < 0.0001.

Vimentin regulates cell mechanics by altering cytoplasmic stiffness (Guo et al., 2013; Vahabikashi et al., 2019). To quantify the changes in the cytoplasmic mechanics of cells expressing WT or ULF mutant vimentin, we probed the intracellular dynamics and mechanics of endocytosed 0.5 µm diameter tracer particles in the cytoplasm by recording spontaneous fluctuations of the particles in living cells. We found that particles inside ULF mutant vimentin cells were less mobile than in the uninduced cells null for vimentin, while particles in the cells expressing WT vimentin were even less mobile (Figure 5A). As expected, the ensemble average of mean square displacement (MSD) of all the particles in different cells exhibited two distinct scaling regimes (Figure 5B): While the low frequency, apparently diffusive regime, is governed by active enzymatic activities, the high frequency regime is dominated by thermal fluctuations (Gupta and Guo, 2017). As shown in Figure 5B, we found that high-frequency (>20 Hz) intracellular dynamics became weaker as vimentin expression was increased, indicating that the cytoplasm was stiffer. At high frequencies spontaneous motion is mainly thermally driven, in which case the modulus is inversely proportional to the average MSD (Mason and Weitz, 1995; Squires and Mason, 2009). Although distinguishable, the relative increase in modulus by expressing vimentin is limited to ∼50% (Figure 5C). Thus, expression of WT or ULF mutant forms of vimentin make the cytoplasm stiffer with WT vimentin having the maximum effect on cytoplasmic stiffness.

### Alterations in gene expression accompany the induction of vimentin expression: *TWIST1* is uniquely upregulated in WT vimentin expressing cells

**FIGURE 6:**
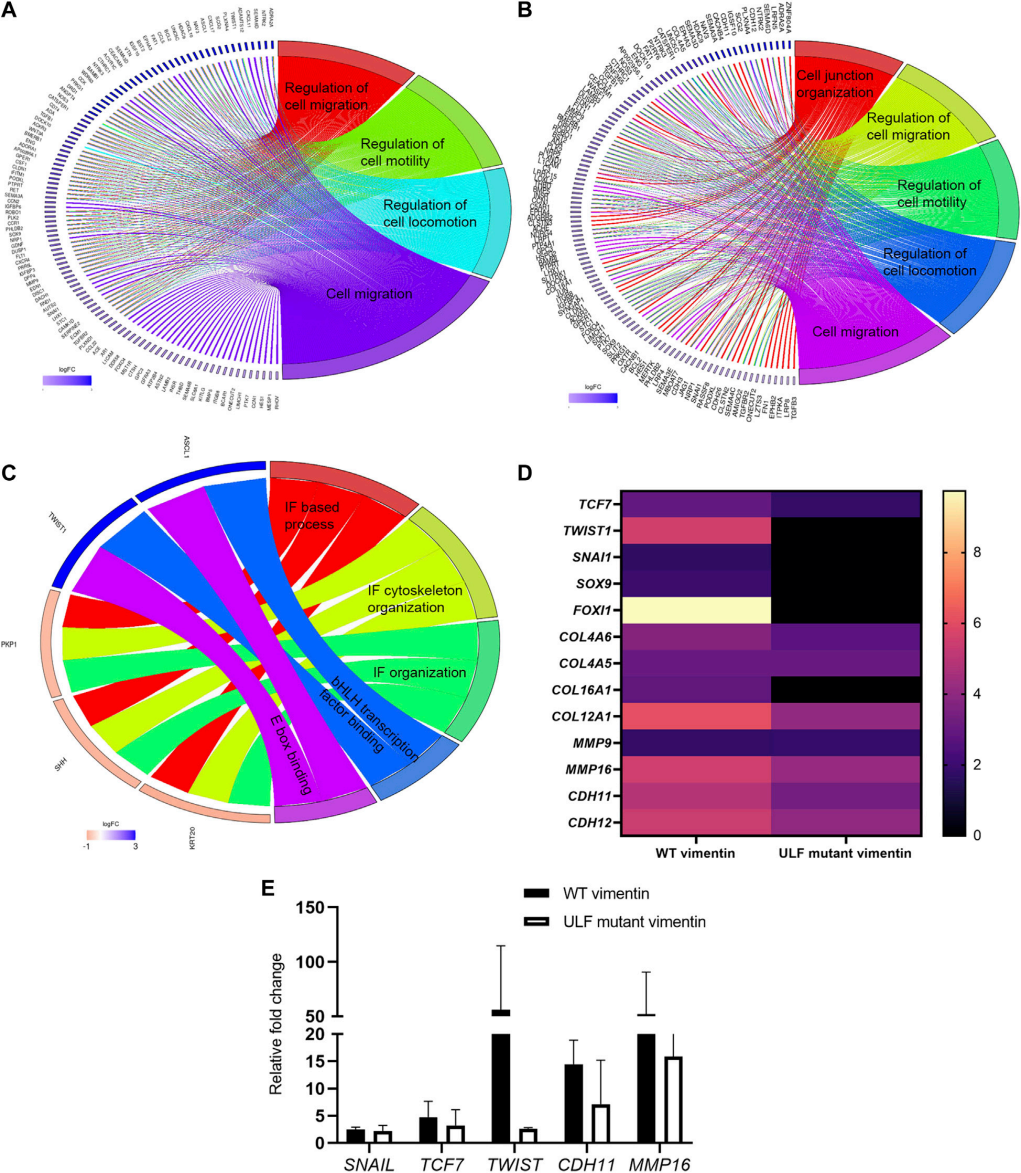
Upregulation of *TWIST1* requires WT vimentin. Circos plot representation of significantly enriched pathways linked with **(A)** WT vimentin vs. uninduced; **(B)** ULF mutant vimentin vs. uninduced; **(C)** WT vimentin vs. ULF mutant vimentin. The ribbon/arc that originates from different genes and terminates at associated pathways demonstrates the connectivity of genes and overrepresented pathways; **(D)** Heatmap shows the fold change values of transcription factors and few of the mesenchymal markers in cells expressing WT or ULF mutant vimentin. **(E)** Graph depicts the relative fold change values of genes obtained by real time PCR (*n* = 3). 18S rRNA was used as the internal control.

To determine if the overall pattern of gene expression changes following the introduction of vimentin into MCF7 epithelial cells during the EMT, we sequenced RNA isolated from MCF7 cells with and without vimentin expression. Differential gene expression analyses identified the increased expression of 481 genes (>2 fold) in WT vimentin expressing cells in comparison with uninduced cells and 159 genes (>2 fold) in ULF mutant vimentin expressing cells. Interestingly, gene set enrichment analysis of the differentially expressed genes indicated that both WT and ULF mutant vimentin activated genes known to be involved in regulating cell locomotion/cell motility and cell junction organization (Figure 6 A and B). To understand how the expression of WT or ULF mutant vimentin impacts these molecular changes, differential gene expression analysis was performed comparing MCF7 cells expressing WT vimentin and those expressing ULF mutant vimentin. There were 78 genes in this category. Strikingly, bHLH transcription factors and E box binding factors were upregulated in cells expressing WT vimentin but not in cells expressing ULF mutant vimentin (Figure 6C). More specifically, *TWIST1*, a major EMT related transcription factor was upregulated in cells expressing WT vimentin, but not in cells expressing ULF mutant vimentin (Figure 6 C and D). Upregulation of *TWIST1* in cells expressing WT vimentin was also confirmed by real time PCR (Figure 6E). Along with the EMT specific transcription factor, *TWIST1,* we also observed an increase in the expression levels of several mesenchymal specific genes such as *CDH11, MMP16, Col12A1* (Figure 6D) some of which are expressed during early hybrid EMT stages (Aiello et al., 2018; Pastushenko and Blanpain, 2019). Hence, our transcriptome analyses suggest that the expression of WT vimentin can induce the expression of the transcription factors necessary for cell migration.

## Discussion

When vimentin expression is induced in MCF7 cells, it first forms particles and then short VIFs or squiggles, which then associate in tandem to form complete VIF networks (Herrmann and Aebi, 2016; Prahlad et al., 1998). We found similar assembly steps in the formation of VIF networks in MCF7 cells following the induction of vimentin expression. With the induction of vimentin expression, we observed that the organization of KIFs is altered with a significant increase in the number of keratin junctions in the cells expressing WT vimentin, indicating a tighter meshwork of KIF. A similar reduction in mesh size or “tightening” of KIF meshworks has been detected in lung alveolar epithelial cells placed under shear stress. This is accompanied by an increase in KIF meshwork stiffness as measured by particle tracking microrheology (Sivaramakrishnan et al., 2008). Based on these findings, we speculate that the organization of KIFs is rearranged in a way to facilitate the stress-enduring capacity of the cells encountered during cell migration following the expression of vimentin and the full transition to a mesenchymal cell phenotype. Based on these considerations, it is possible that the tighter meshworks formed by the bundles of KIFs (tonofibrils) work synergistically with the induction of vimentin to increase the stiffness of MCF7 cells.

Coincident with the changes in vimentin expression and assembly, desmosomes are disrupted in the MCF7 cells. The importance of desmosomes in cancer progression has been shown in the conditional knock-out of the *Dsp* gene in mouse pancreatic β cells where loss of desmosomes increased local invasion, even though the cells retained intact E-cadherin expression (Chun and Hanahan, 2010). Similarly, the loss of the desmosomal protein *Perp* has been suggested to be involved in epithelial cancer progression, under conditions where adherens junctions are not affected (Beaudry et al., 2010). Based on these studies, it has been suggested that epithelial cancer progression occurs in two steps; with desmosomal loss being the initial step followed by the loss of adherens junctions (Dusek and Attardi, 2011). In our study, we have observed the mislocalization of desmoplakin and disruption of desmosomes in cells expressing WT vimentin and to a lesser extent by ULF mutant vimentin. However, our transcriptome analysis indicates that the expression of the adherens junction marker, *E-cadherin,* is not altered upon expression of vimentin. This is supported by our analysis of the localization of E-cadherin in uninduced and in WT vimentin or ULF mutant vimentin expressing MCF7 cells (Supplementary Figure S5). With these observations, we can speculate that the expression of vimentin regulates the initial steps of the EMT associated with cancer metastasis/progression.

Our results also demonstrate that the expression of both WT and ULF mutant vimentin reduce traction and intercellular forces. In support of this finding, osteosarcoma cells expressing KIFs but lacking VIFs exert increased traction forces compared to these cells when vimentin is expressed (Jiu et al., 2017). In addition, the disruption of desmosome-KIF interactions by the expression of a dominant negative mutant of desmoplakin decreases both cell-cell and cell-substrate forces in epithelial carcinoma cells. There is also an increase in the cell-cell and cell-substrate forces when the desmosome-KIF interaction is strengthened (Broussard et al., 2017). Based upon these findings, it is possible that the induction of vimentin expression and its polymerization into VIFs or ULFs in MCF7 cells, may act to displace desmoplakin-KIF interactions and thereby reduce the intercellular and cell-substrate forces that we detected in our study. Other possible reasons for the ULF mutant vimentin exerting lesser traction force on the substrate could be due to changes in focal adhesion composition and organization (see Supplementary Figure S6) including reduced linkages of KIFs to the desmoplakin in the cells expressing ULF vimentin.

The impact of the expression of VIFs on cortical and cytoplasmic stiffness has been shown in mesenchymal cells (Guo et al., 2013; Vahabikashi et al., 2019). Cells expressing vimentin are twice as stiff as vimentin null cells which contain no cytoskeletal IFs (Guo et al., 2013). In further support of this we find that in MCF7 cells expressing either the WT or ULF mutant forms of vimentin have reduced intracellular dynamics assayed by the movements of endocytosed particles, indicating an increase cytoplasmic stiffness. It has been shown that the expression of vimentin and the metastatic potential of cancerous cells are correlated (Wei et al.,2008). Therefore, it is possible that our findings showing an increased cytoplasmic stiffness in cells expressing vimentin may be involved in the induction of cell migration and metastasis. Magnetic resonance elastography performed on breast and liver tumors to evaluate the stiffness of the tumors has revealed that malignant tumors are stiffer than benign tumors and normal tissues (Lorenzen et al., 2002; Venkatesh et al., 2012). In addition, cell stiffness increases with an increase in invasive potential in human breast carcinoma and glioblastoma cells (Kim et al., 2016; Monzo et al., 2021). In contrast, there is a widespread notion that cells with higher metastatic potential are more deformable and compliant (Holenstein et al., 2019; Plodinec et al., 2012; Swaminathan et al., 2011). In order to explain these differences, it may be that the relationships between cell stiffness and metastatic potential are cell type specific and/or vary based on the methods used for measuring stiffness. Another factor in these differences may be related to the “tuning” of the mechanical properties of cancerous cells at different stages of metastasis (Gensbittel et al., 2021).

Expression of WT and ULF mutant vimentin in MCF7 cells activates pathways involved in cell migration and cell junction organization. Interestingly, upregulation of *TWIST1*, an EMT specific transcription factor is seen only in cells which express WT vimentin, and not ULF mutant vimentin. *TWIST1* is involved in all the stages of cancer metastasis and its expression contributes to metastasis and invasion by inducing the EMT (Yang et al., 2004). Moreover, TWIST1 regulates the expression of vimentin during the EMT by increasing the expression of Cul2 circular RNA (Meng et al., 2018). Since the upregulation of *TWIST1* was observed only in WT vimentin expressing cells, we speculate that fully polymerized VIFs, but not partially assembled ULF could influence the regulation of *TWIST1.* This latter finding may be related to the role of polymerized VIF networks in signal transduction (Chang and Goldman, 2004; Helfand et al., 2005). In further support of this possibility, vimentin is known to act as a scaffold for kinases such as ERK and it recruits proteins for phosphorylation (Perlson et al., 2006; Virtakoivu et al., 2015). In addition, it has been shown that TWIST1 is regulated by ERK kinase (Weiss et al., 2012) which in turn is regulated by vimentin (Perlson et al., 2006; Virtakoivu et al., 2015). In the future it will be interesting to determine if VIFs, but not ULFs, act as a scaffold for certain kinases such as ERK, which might result in the upregulation of *TWIST1.*

Although we have observed an upregulation of *TWIST1* and several other mesenchymal markers such as *CDH11, MMP16, MME* and collagens upon the induction of vimentin expression, we did not see changes in the expression of E*-cadherin*, an important marker for the EMT transition. Recently, a partial or hybrid EMT has been identified in which E-cadherin and the expression of epithelial markers such as *Epcam*, *Cldn2* and *Cldn4* are retained but cells still undergo a successful metastasis (Aiello et al., 2018). Cells undergoing a partial EMT possess both epithelial and mesenchymal characteristics, which have been related to collective migration (Aiello et al., 2018; Pastushenko and Blanpain, 2019). Also, whether the cells undergo a partial or complete EMT seems to depend on the subtype of tumors. Interestingly, MCF7 cells are predicted to go through a partial EMT process for cancer dissemination (Aiello et al., 2018). In agreement with this observation, our results in MCF7 cells show no changes in the expression levels of *E-cadherin,* but do appear to show an upregulation of other mesenchymal markers associated with a hybrid EMT. Based on these results, we hypothesize that the expression of WT vimentin drives the MCF7 cells into early/late partial EMT stages.

ULF–Unit Length Filament; VIF–Vimentin Intermediate Filament; KIF–Keratin Intermediate Filament; EMT–Epithelial to Mesenchymal Transition; SIM–Structured Illumination Microscopy; MSD–Mean Square Displacement.

## Data Availability

The datasets presented in this study can be found in online repositories. The names of the repository/repositories and accession number(s) can be found below: Gene Expression Omnibus accession number: GSE206724.
