# Supplementary material for: Expression of vimentin alters cell mechanics, cell-cell adhesion, and gene expression profiles suggesting the induction of a hybrid EMT in human mammary epithelial cells
Source: Front Cell Dev Biol. 2022 Sep 19;10:929495. doi: 10.3389/fcell.2022.929495 (PMC9527304; doi:10.3389/fcell.2022.929495)
Supplement: Supplementary file 1 [file Image1.pdf]

Supplementary figures

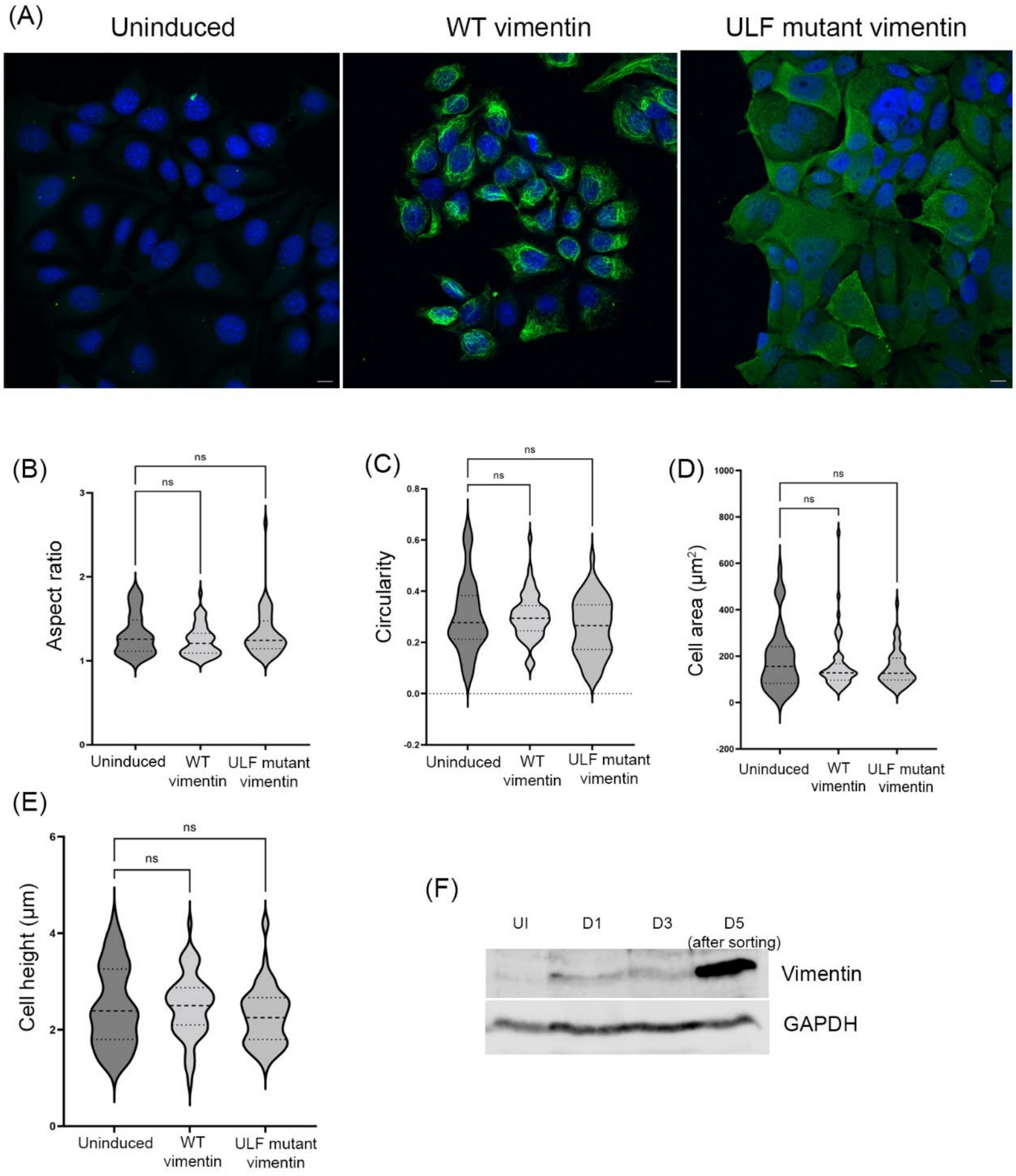

Supplementary figure 1: Immunofluorescence images of (A) Uninduced, WT vimentin and ULF mutant vimentin showing that vimentin is expressed in the majority of induced cells. Scale bar is 10μm. Violin plots showing the (B) aspect ratio (C) circularity (D) cell area and (E) cell height in these groups of cells. (F) Western blot showing the expression of vimentin in cells induced to express WT vimentin on days 1, 3 and 5 post induction (after sorting) with cumate.

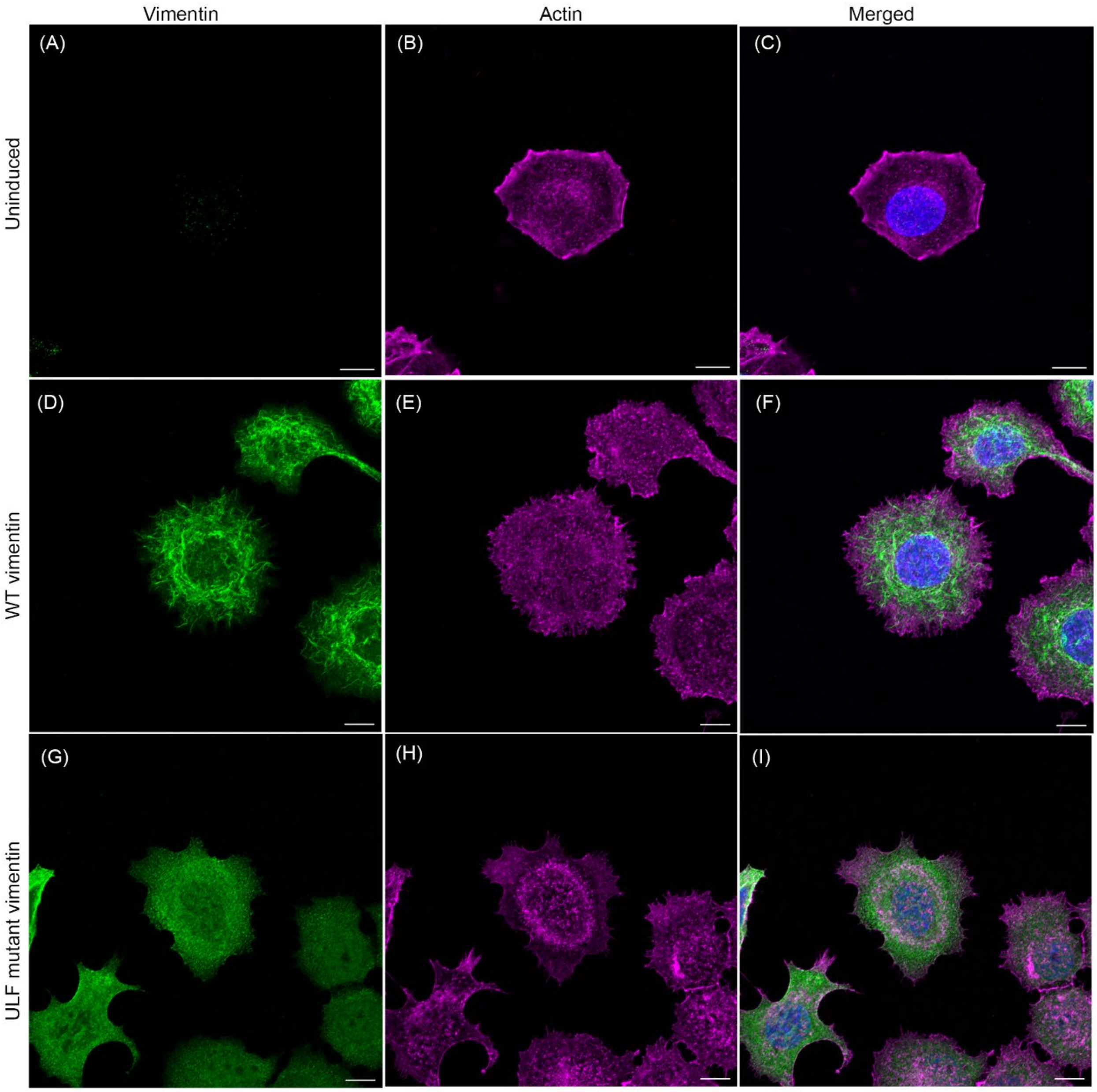

Supplementary figure 2: Immunofluorescence images showing vimentin and actin organization in uninduced (A-C), and in WT (D-F) and ULF mutant vimentin (G-I) expressing cells. Nucleus is stained with Hoechst. Scale bar is 10μm.

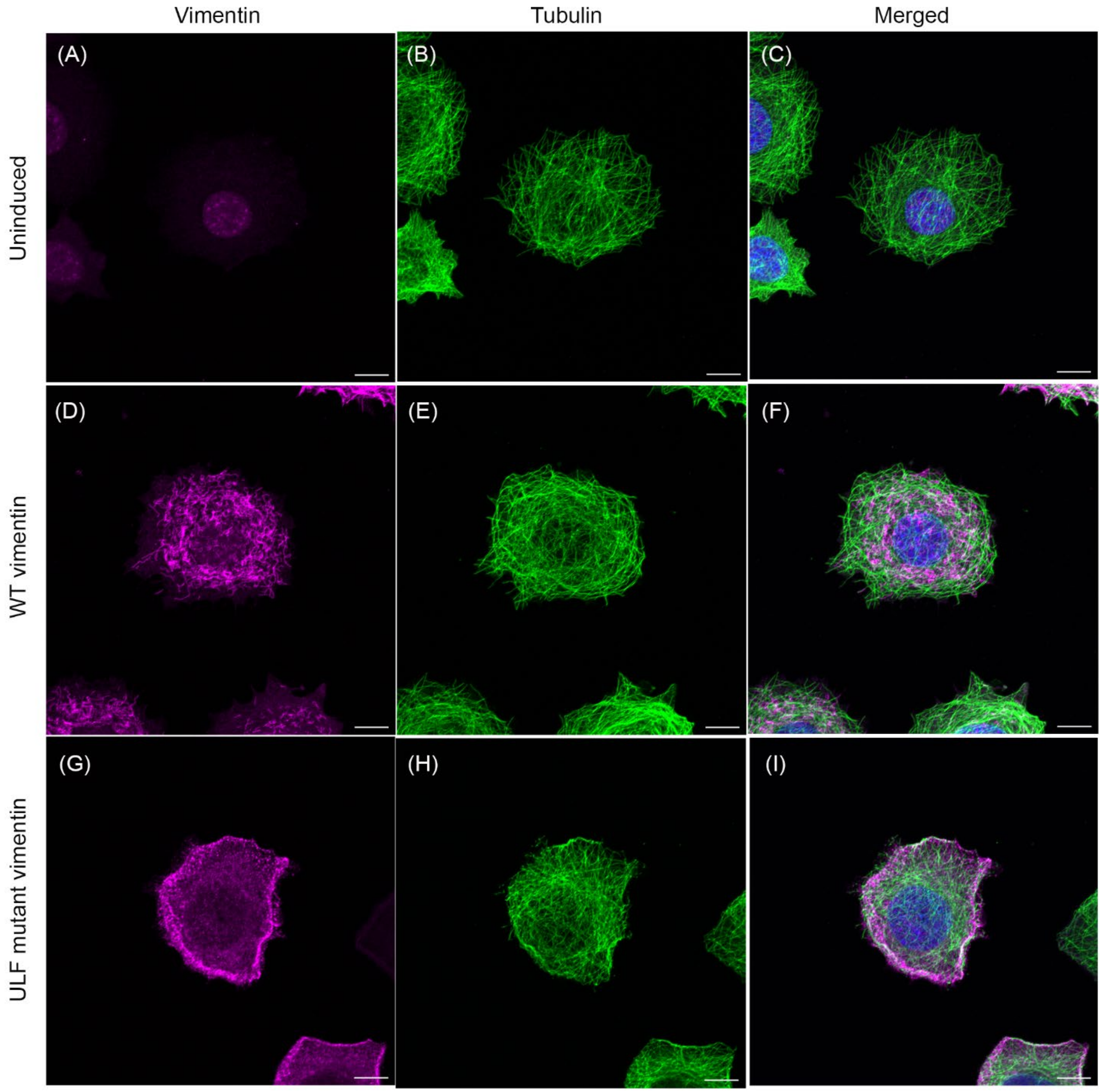

Supplementary figure 3: Immunofluorescence images showing vimentin and microtubule organization in uninduced (A-C), and in WT (D-F) and ULF mutant vimentin (G-I) expressing cells. Nucleus is stained with Hoechst. Scale bar is 10μm.

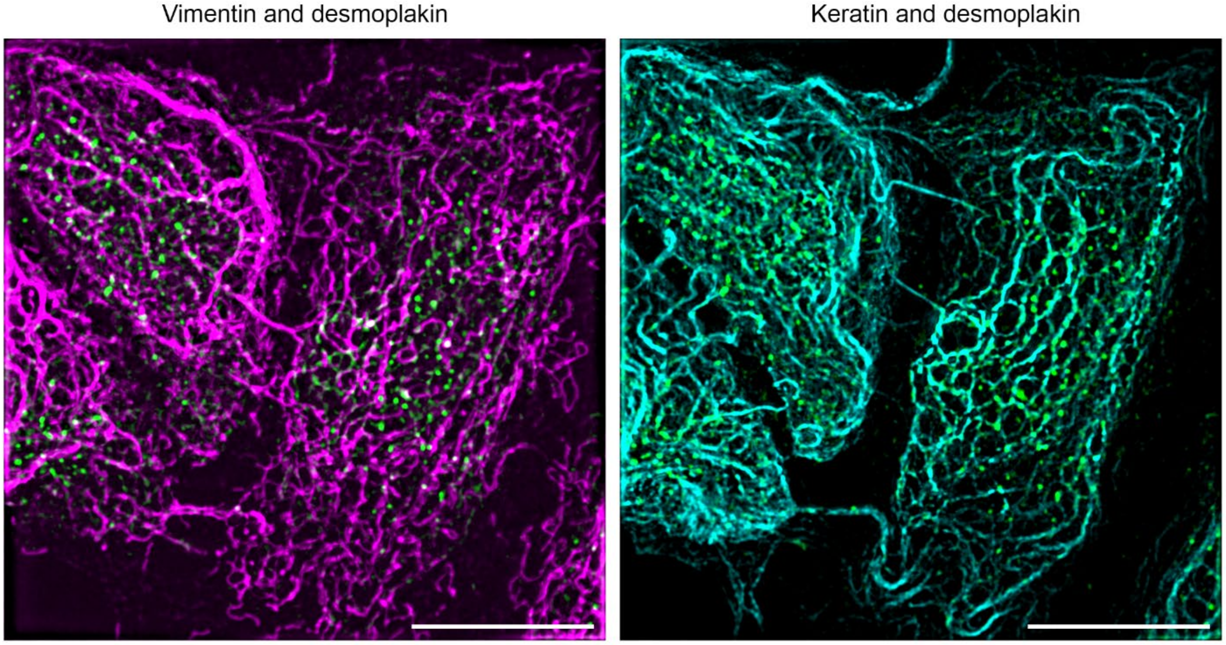

Supplementary figure 4: Immunofluorescence images showing the association of VIFs (magenta) and desmoplakin (green); KIF (cyan) and desmoplakin (green) in MCF7 cells induced to express WT vimentin. Scale bar is 10µm.

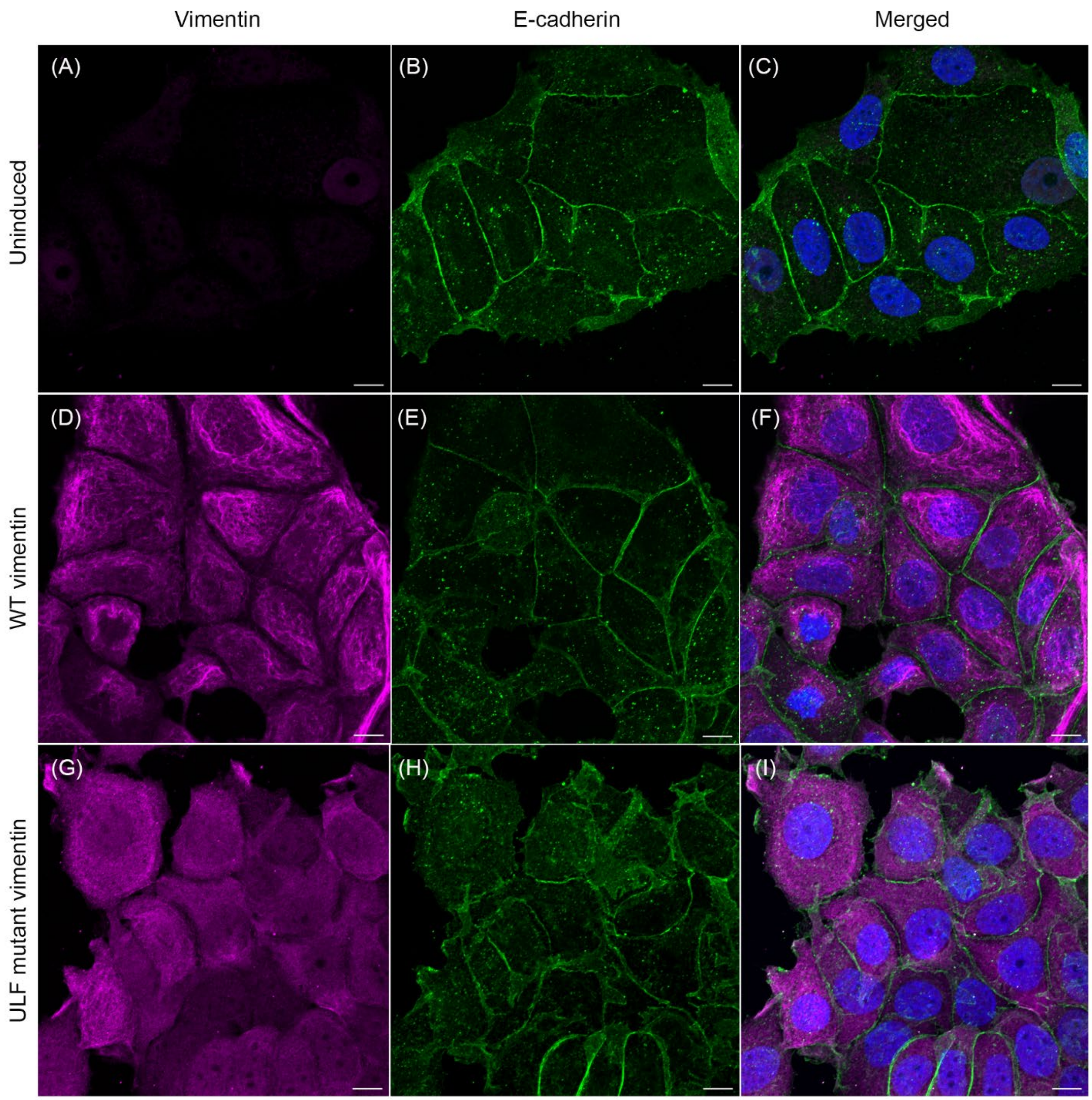

Supplementary figure 5: Immunofluorescence images showing vimentin and E-cadherin in uninduced (A-C), and in WT (D-F) and ULF mutant vimentin (G-I) expressing cells. Nucleus is stained with Hoechst. Scale bar is 10µm.

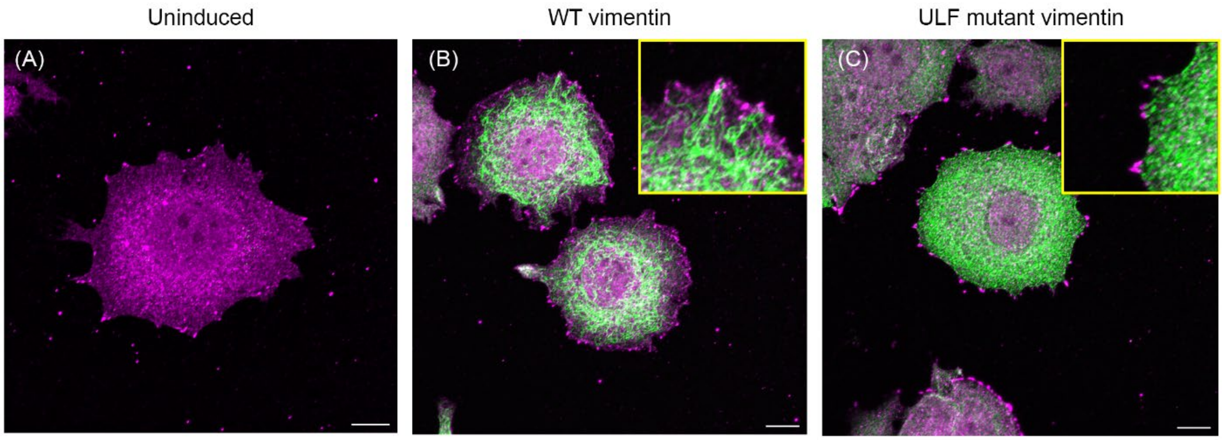

Supplementary figure 6: Immunofluorescence images of Uninduced (A), WT vimentin (B) and ULF mutant vimentin (C) showing vimentin (green) and vinculin (magenta). Inset shows a magnified region of the cell periphery. Note the close approximation of vimentin IFs to vinculin containing focal adhesions. Scale bar is 10μm.

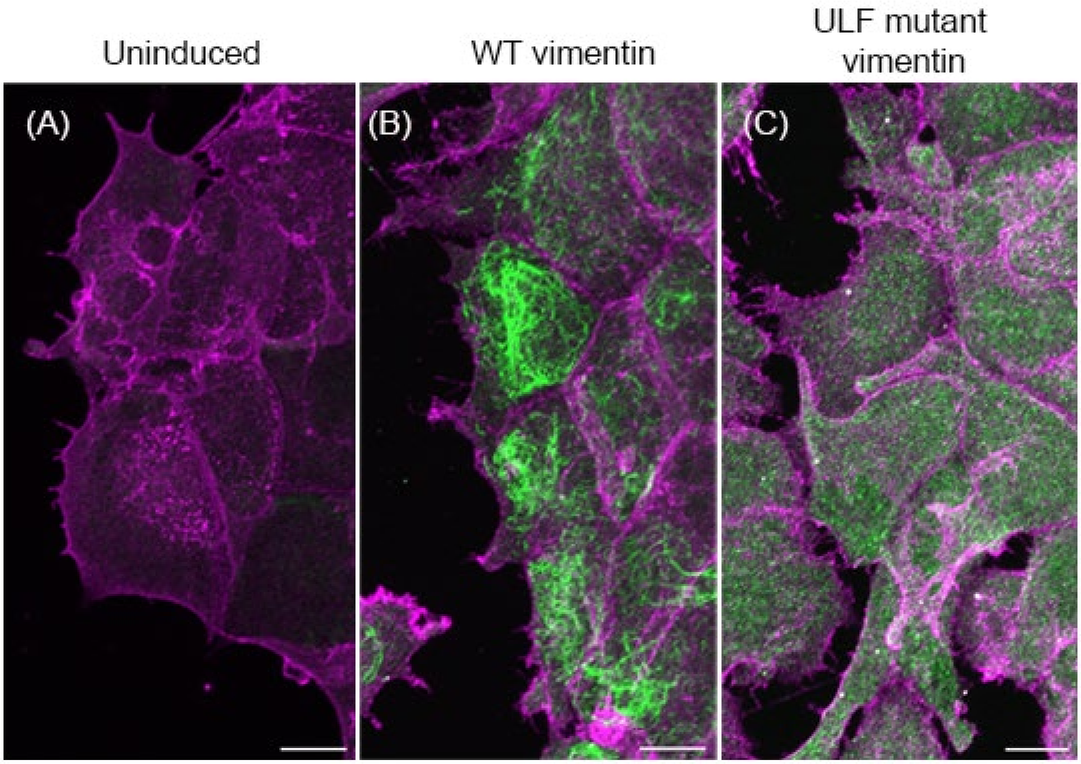

Supplementary figure 7: Immunofluorescence images of uninduced (A), WT vimentin (B, green) and ULF mutant vimentin (C, green) and β1 integrin (magenta). Scale bar is 10μm.
